# Long-latency auditory evoked response amplitudes at first episode of psychosis predict six-month recovery in positive symptom severity

**DOI:** 10.1016/j.psychres.2024.116094

**Published:** 2024-07-21

**Authors:** Brian A. Coffman, Mark Curtis, Dylan Seebold, Jenay Kocsis, Aseem Dani, Fran López-Caballero, Alfredo Sklar, Dean F. Salisbury

**Affiliations:** Clinical Neurophysiology Research Laboratory, Western Psychiatric Hospital, Department of Psychiatry, University of Pittsburgh School of Medicine, Pittsburgh, PA, USA

## Abstract

Predicting treatment response would facilitate individualized medical treatment in first-episode psychosis (FEP). We examined relationships between auditory-evoked M100 and longitudinal change in positive symptoms in FEP. M100 was measured from source-resolved magnetoencephalography and symptoms were assessed at initial contact and six months later. M100 at baseline significantly predicted symptom change. Larger M100 at baseline predicted symptom improvement, as did shorter untreated psychosis. Shorter untreated psychosis also correlated with larger M100, and M100 mediated the effect of untreated psychosis on treatment response. Thus, M100 may provide a proximal and objective index of untreated psychosis and a viable route to individualized medicine.

## 1. Introduction

Auditory hallucinations (AH) are common symptoms of psychosis and often prompt clinical contact. Despite pharmacotherapy, about 25 % of individuals continue to experience positive symptoms (Shergill et al., 1998), impacting long-term outcome (Emsley et al., 2007). Currently, there are no established methods to classify individuals likely to respond to standard treatments. Studies of temporal lobe dysfunction in early stages of the disorder may provide insight into the biological mechanisms underlying treatment response variability; recovery of auditory neurophysiology is related to symptom recovery over time (Coffman et al., 2023), functional connectivity between auditory cortex and medial temporal lobe predicts AH symptom response to treatment (Anhøj et al., 2023), and the duration of untreated psychosis may be related to temporal lobe dysfunction early in the disorder (Gonzalez-Heydrich et al., 2015) (Fig. S1).

Untreated psychosis is known to mediate antipsychotic treatment response but does not correlate with positive symptoms at initial treatment (Penttilä et al., 2014), suggesting that untreated psychosis (much like M100) predicts treatment-related symptom changes, rather than functional outcomes more generally (Perkins et al., 2005). However, mechanisms of potential “neurotoxic” effects of untreated psychosis are elusive. Although recent meta-analysis refutes the notion of psychosis as structurally or functionally neurotoxic at the whole-brain level, evidence for temporal lobe neuropathy remains (Zoghbi et al., 2023). The duration of untreated psychosis (DUP) is defined as the time from first symptom to first treatment; however, the commonly-experienced prodromal period prior to first psychosis (Fusar-Poli et al., 2011) makes symptom onset identification somewhat subjective (Singh, 2007). Medical records and family member interviews can be used to supplement symptom onset date by identifying the first observable active psychotic episode – the duration of untreated active psychosis (DAP). The lack of distinction between DUP and DAP may be one source of variability in untreated psychosis research (Fig. S1).

In this study, we build upon our prior report of concomitant changes in M100 and symptoms by investigating baseline M100 as a potential product of untreated psychosis and predictor of symptom change. We hypothesized that M100 amplitudes at study entry would predict treatment response, untreated psychosis would predict M100 amplitudes at initial treatment, untreated psychosis would predict treatment response for positive symptoms, and M100 amplitudes would mediate the relationship between untreated psychosis and treatment response for AH. Symptom change was modeled as a function of baseline severity, untreated psychosis duration, and M100 amplitudes. Odds ratios were calculated for AH remission given M100 amplitude and initial AH presence.

## 2. Methods

### 2.1. Participants

Participants were 24 FEP recruited from Western Psychiatric Hospital (for demographic data, see Table S1). Symptom severity over the prior 2-weeks was rated using the PANSS and Psychosis Symptom Rating Scales (PSYRATS). Structured interviews (SCID) and detailed medical record follow-back were used to obtain dates of symptom onset, active psychosis onset (record of psychosis symptoms recognized by a medical professional), and treatment onset (first day of antipsychotic treatment or day of M100 data collection, if untreated) (see Fig. S1). Most participants were medicated at baseline and follow-up (Table S2). Psychosis onset dates were determined more reliable than symptom onset dates (Fig. S2). Thus, the number of weeks from active psychosis onset to first treatment (DAP) was used to measure untreated psychosis in this study. All interviews and tests were overseen by a Masters’- or PhD-level clinical psychologist. Participants provided informed consent and were paid for participation.

### 2.2. Procedures

Magnetoencephalography (MEG) data were recorded with a 306-channel whole-head system (Elekta Neuromag) in a magnetically shielded room while simple tones (50ms duration, 10ms rise/fall) were presented in an “*Oddball*” design, with 340 standard tones (1kHz), 60 deviant tones (1.2 kHz), and stimulus onset asynchrony of 1050-1550ms. Participants were instructed to ignore tones and attend a silent video. MEG data were source-resolved to auditory cortex ROIs (left/right A1, lateral belt, and parabelt), and signed dSPM value was averaged across ROI vertices over the 80-140 ms time window as in (Curtis et al., 2022).

### 2.3. Data Analysis

Sequential linear regression was used to test whether M100 amplitude at initial evaluation improved prediction of change in symptom severity beyond what was predicted by the baseline measurement (i.e., regression to the mean). We then tested whether M100 mediates relationships between DAP and symptom change, controlling for baseline severity. Logarithmic transformation of DAP, treatment duration, and total antipsychotic medication was used to improve normality/linearity, and to reduce the impact of outliers on residuals. We additionally performed categorical assessment of auditory cortex M100 waveforms. Using this approach, we calculated odds ratios for response to treatment given auditory cortex M100 amplitude <= −0.05 dSPM in either hemisphere. Response to treatment was defined as no AH at follow-up (PSYRATS AH score = 0). Odds ratios were calculated across all subjects (*N* = 24) and for the subset of subjects who reported AH symptoms > 0 at baseline (*N* = 13).

## 3. Results

**Fig. 1.**
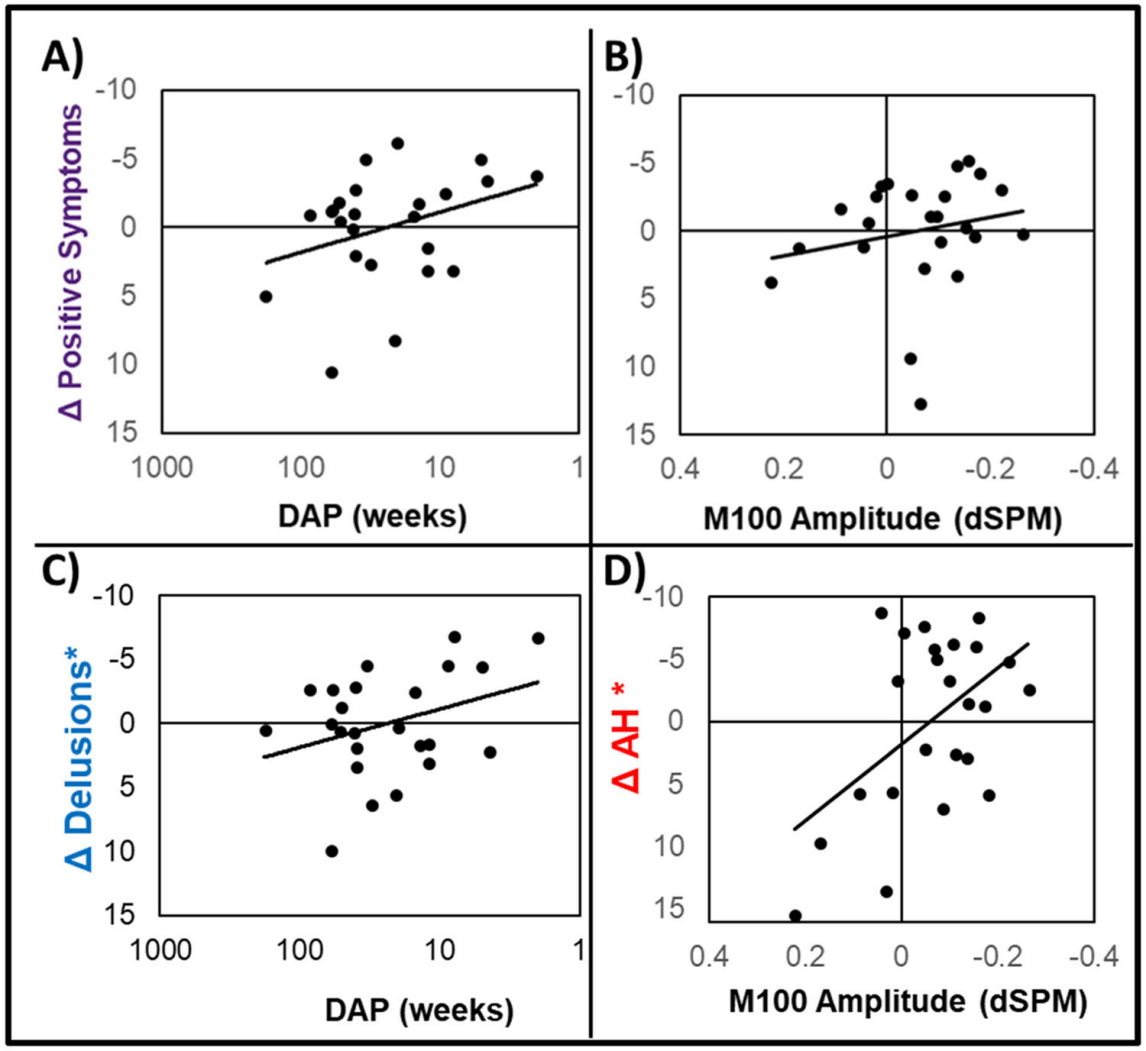
Scatterplots are shown for independent effects of M100 amplitude (**A**) and DAP (**B**) on change in positive symptoms measured with the PANSS, controlling for the effects of positive symptom scores at baseline. Panel **C** depicts the effect of DAP on change in delusions measured with the PSYRATS, controlling for the effects of baseline delusions and M100 amplitude, indicating DAP independently predicts course of delusions in this sample. Panel **D** shows the effect of M100 amplitude on change in AH measured with the PSYRATS, controlling for the effects of baseline AH and DAP, indicating M100 amplitudes predict course of AH in this sample. Axes for all scatterplots are oriented with more severe/undesirable scores at the lower left.

Individual waveforms are shown in Figs. S3/S4. Symptoms improved over time at the group level (Table S2). Symptom improvement was unrelated to medication dosage at baseline (*p*’s>0.1) and was related to reduced total medication in the interim between assessments (*r* = 0.42, *p* < 0.05). Longer DAP (log transformed) was correlated with reduced M100 at baseline (*r* = 0.49; *p* = 0.015) and reduced PANSS positive symptoms change (*r* = 0.45; *p* = 0.029). Baseline PANSS positive symptoms accounted for 28 % of the variance in positive symptom improvement (*F*_(1,22)_=8.55; *p* < 0.01; Table S3), where those with greatest initial symptom severity showed larger reductions in symptoms. This relationship was not suppressed by adding M100 or DAP into the model; however, adding either M100 or DAP significantly increased R^2^, indicating both M100 and DAP predict positive symptom change beyond what can be expected by regression to the mean. Results from the full model indicate that M100 and DAP may have bidirectional mediation effects, as both β statistics decreased in this model, while R^2^ significantly increased (Fig. 1).

Baseline symptom scores accounted for 66 % of the variance in AH symptom change (*F*_(1,22)_=43.66; *p* < 0.001; Table S4) and 43 % of the variance in delusion change (*F*_(1,22)_=16.39; *p* < 0.001; Table S5). This relationship was not suppressed by M100 or DAP; however, M100 added only 8 % variance in predicting delusions (*F*_(1,21)_=3.46; *p* = 0.07) while DAP contributed 15 % variance to the model (Fig. 1). Further, adding M100 did not meaningfully change model fit or the β statistic for DAP. Conversely, M100 and DAP both improved prediction of AH, where M100 amplitude increased R^2^ to a greater degree than DAP (ΔR^2^ = 0.13/0.08, respectively). Most importantly, adding M100 to the final model increased R^2^ and reduced the β statistic for DAP by nearly 50 %, eliminating its direct effect on AH change. Thus, auditory cortex M100 amplitude mediates of the effect of DAP on AH symptom change, accounting for approximately 12 % variance (Fig. 1).

Seventeen participants had M100 amplitudes larger than −0.05 dSPM in at least one hemisphere and were therefore not predicted to have AH at follow-up (Fig. S3), while the remaining 7 were predicted to have residual AH symptoms (Fig. S4). M100 significantly predicted residual AH (O*R* = 11.67; 95 % CI=9.61-13.73). When restricted to participants with non-zero AH scores at baseline (7 predicted to have no AH, 6 predicted to have residual symptoms), this effect remained relatively unchanged (O*R* = 12.00; 95 % CI = 9.29-14.71).

## 4. Discussion

In this study of first-episode psychosis, healthier M100 responses at baseline predicted better symptom improvement after ~6 months. Conversely, those with impaired M100 at baseline improved less with frontline treatment. Further, untreated psychosis (longer DAP) predicted reduced positive symptom change and was related to reduced M100, indicating untreated psychosis negatively impacted temporal lobe function. Effects of untreated psychosis on AH treatment response were fully mediated by M100; those with longer DAP had smaller M100 amplitude, which then predicted reduced treatment response. However, this wasn’t the case for delusions, where DAP effects were independent of M100 amplitude.

Although sample size here was small (*N* = 24), and results are preliminary, this is the first study to draw a link between untreated psychosis, temporal lobe function, and AH response to treatment. Efforts to improve antipsychotic medication effectiveness would benefit from reliable biomarkers that predict treatment response. Pharmacogenetic studies have identified variants of the DRD_2_ and COMT genes as potential predictors of antipsychotic treatment response; however, these effects are small (Zhang and Malhotra, 2017). Structural neuroimaging biomarkers have also been proposed, but lack potential for tracking treatment effectiveness, as changes are delayed. Thus, functional neurophysiological biomarkers such as the M100 would provide greater translational impact than genetic or structural markers.

The experience of auditory hallucinations is one of the most distressing features of psychosis. Our results suggest M100 responses may identify individuals who would benefit from targeted intervention. Individuals with healthier M100 responses showed better symptom improvement, while those with reduced M100 might benefit from more aggressive interventions. M100 could provide immediate information about the appropriate level of intervention at baseline. Odds ratios determining AH remission from M100 amplitudes were large, and only increased when considering baseline symptoms as well as M100 amplitudes. Thus, measurement of auditory neurophysiological function provides a viable route to individualized medicine.

## Supplementary Material

Supplementary Materials
