# Supplementary material for: Long-latency auditory evoked response amplitudes at first episode of psychosis predict six-month recovery in positive symptom severity
Source: Psychiatry Res. Author manuscript; Available in PMC 2024 Sep 1. (PMC11321909; doi:10.1016/j.psychres.2024.116094)
Supplement: Supplementary Materials [file NIHMS2012034-supplement-Supplementary_Materials.docx]

**Supplemental Materials for “Long-Latency Auditory Evoked Response Amplitudes at First Episode of Psychosis Predict Six-Month Recovery in Positive Symptom Severity”**

**
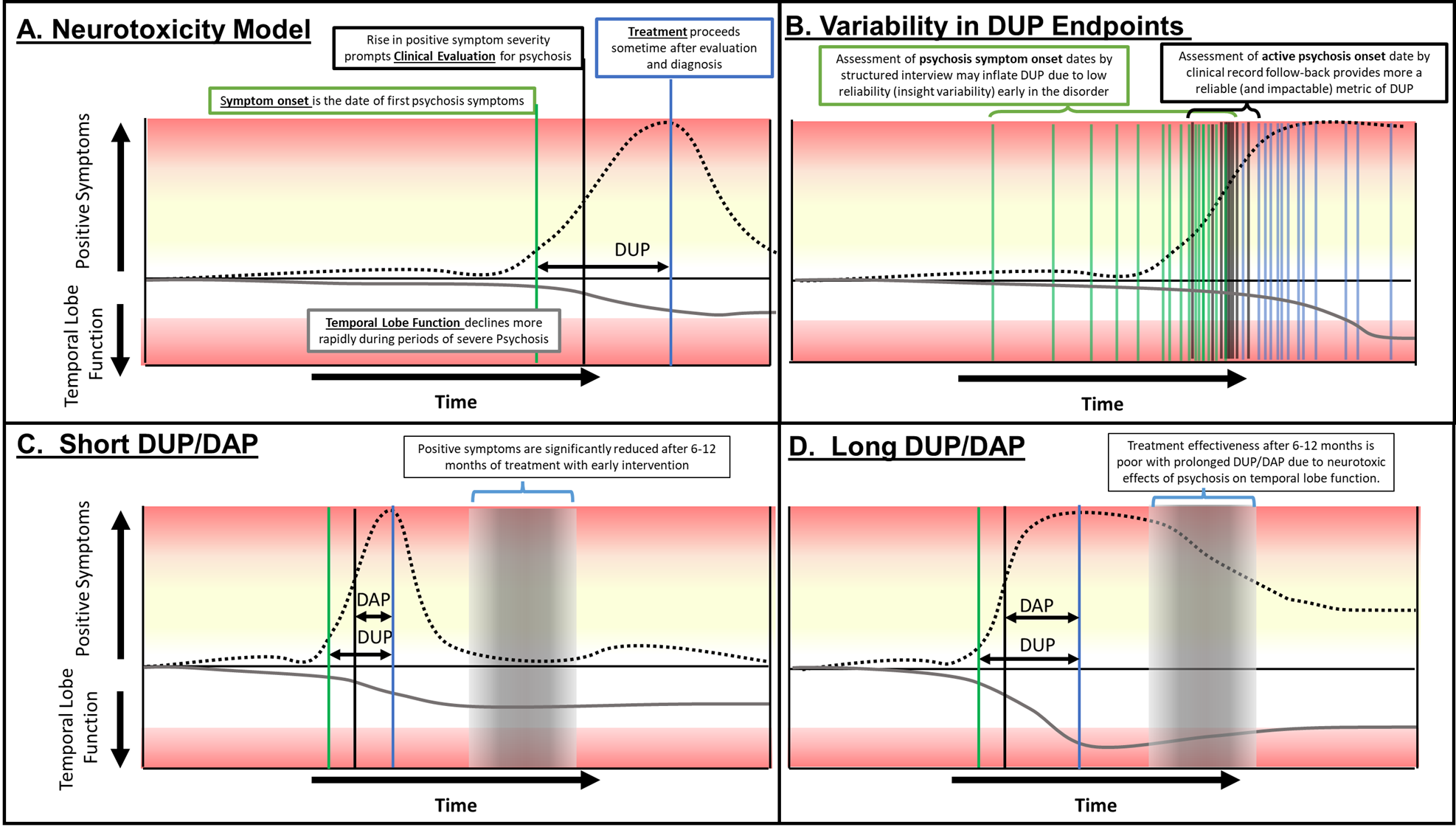
**

**Figure S1.** Graphical depiction of how DUP/DAP may impact positive symptom recovery via accelerated decline in temporal lobe function during the period of untreated psychosis. The neurotoxicity model of psychosis is shown in **Panel A**, with symptom onset, active psychosis onset, and treatment onset endpoints defined. **Panel B** depicts variability commonly identified for each of these endpoints and highlights difficulties with assessment of symptom onset dates. With short DUP/DAP (**Panel** **C**), temporal lobe function does not decline to a level that hinders treatment effectiveness (represented by red shading at the bottom of the graph); however, longer DUP/DAP (**Panel D)** results in severe decline of function in the temporal lobe and greater difficulty in reducing symptoms with treatment. Grey shading represents times at which M100 amplitudes and symptoms were measured in the longitudinal study from which these data are drawn (Coffman et al., 2023).

**
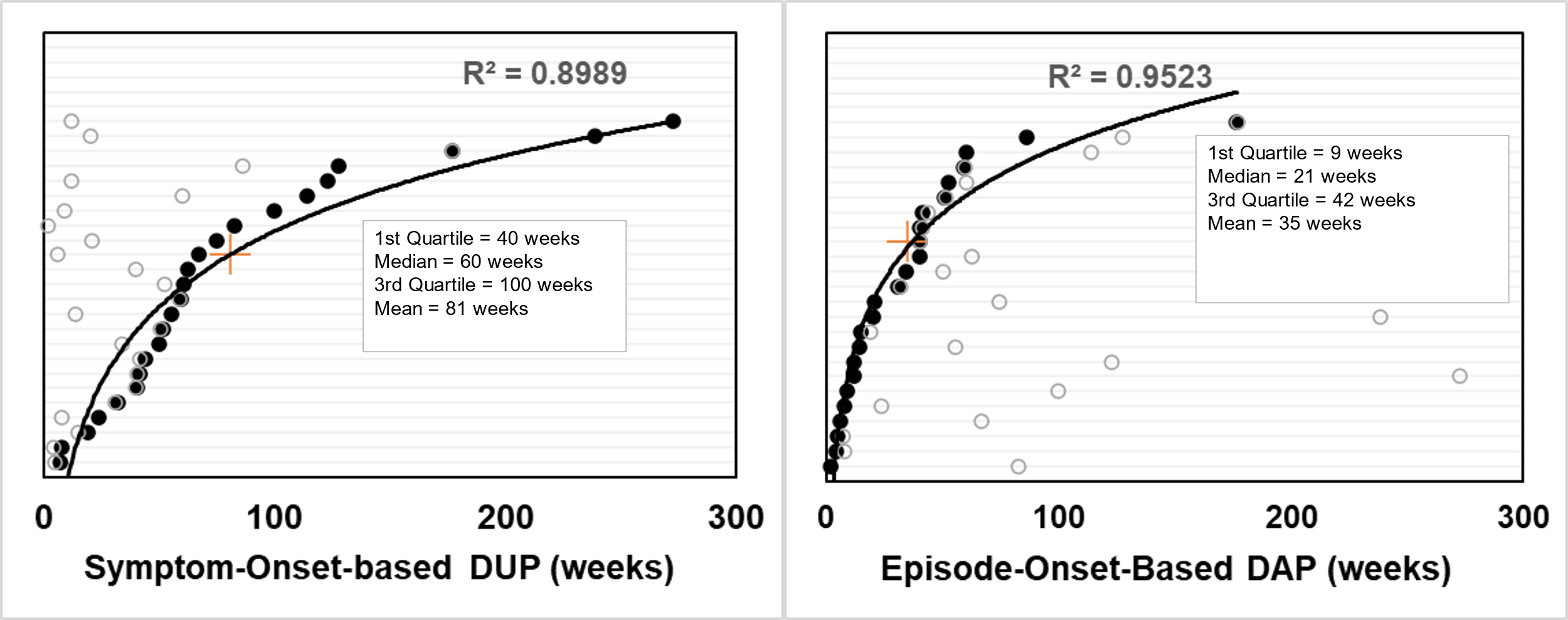
**

**Figure S2.** Distributions of DUP (left) and DAP (right) in weeks to treatment are depicted by filled circles with fitted black regression lines. Unfilled circles represent the corresponding unsorted DAP (left) and DUP (right) values.

**Figure S3.**


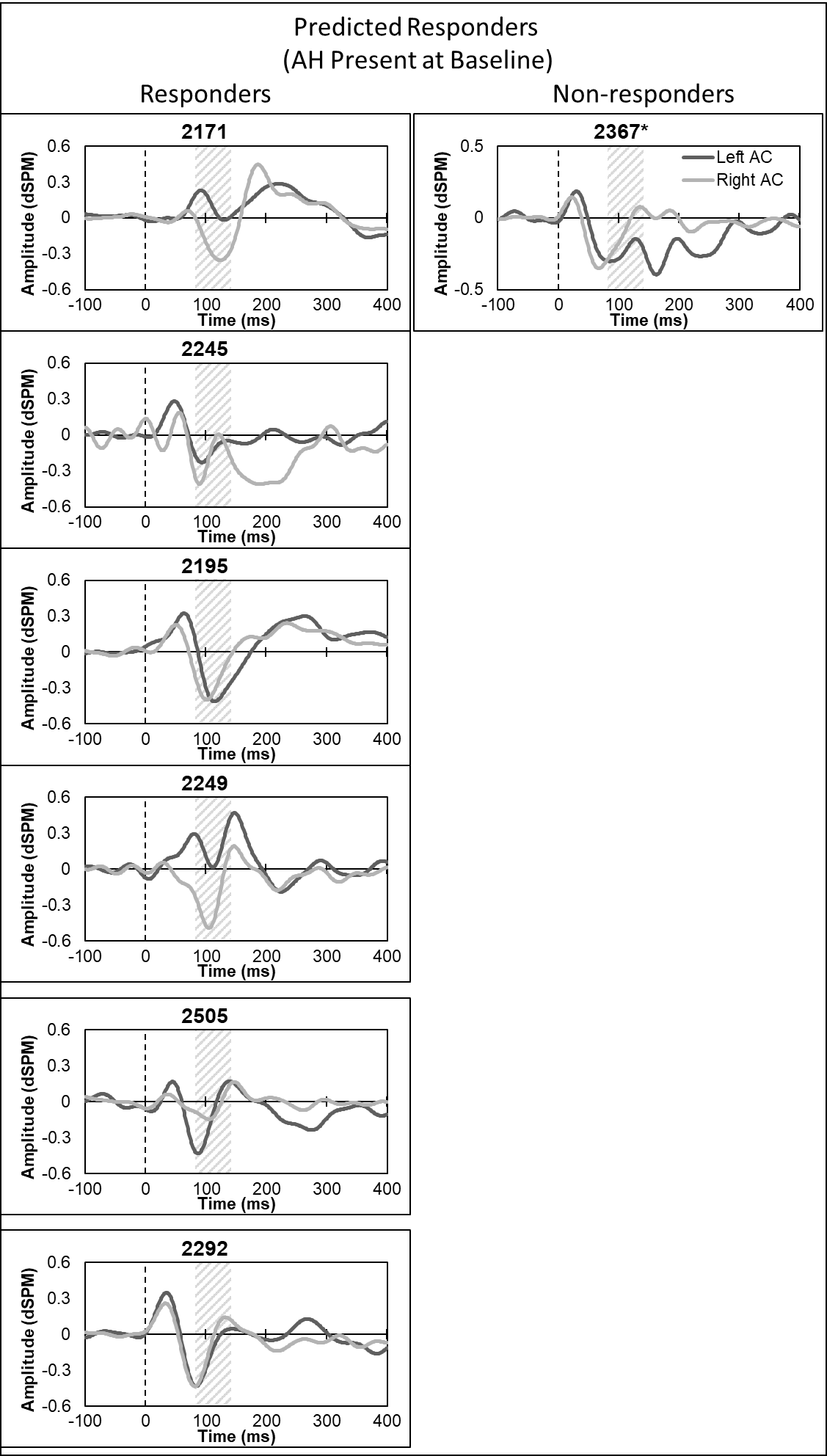

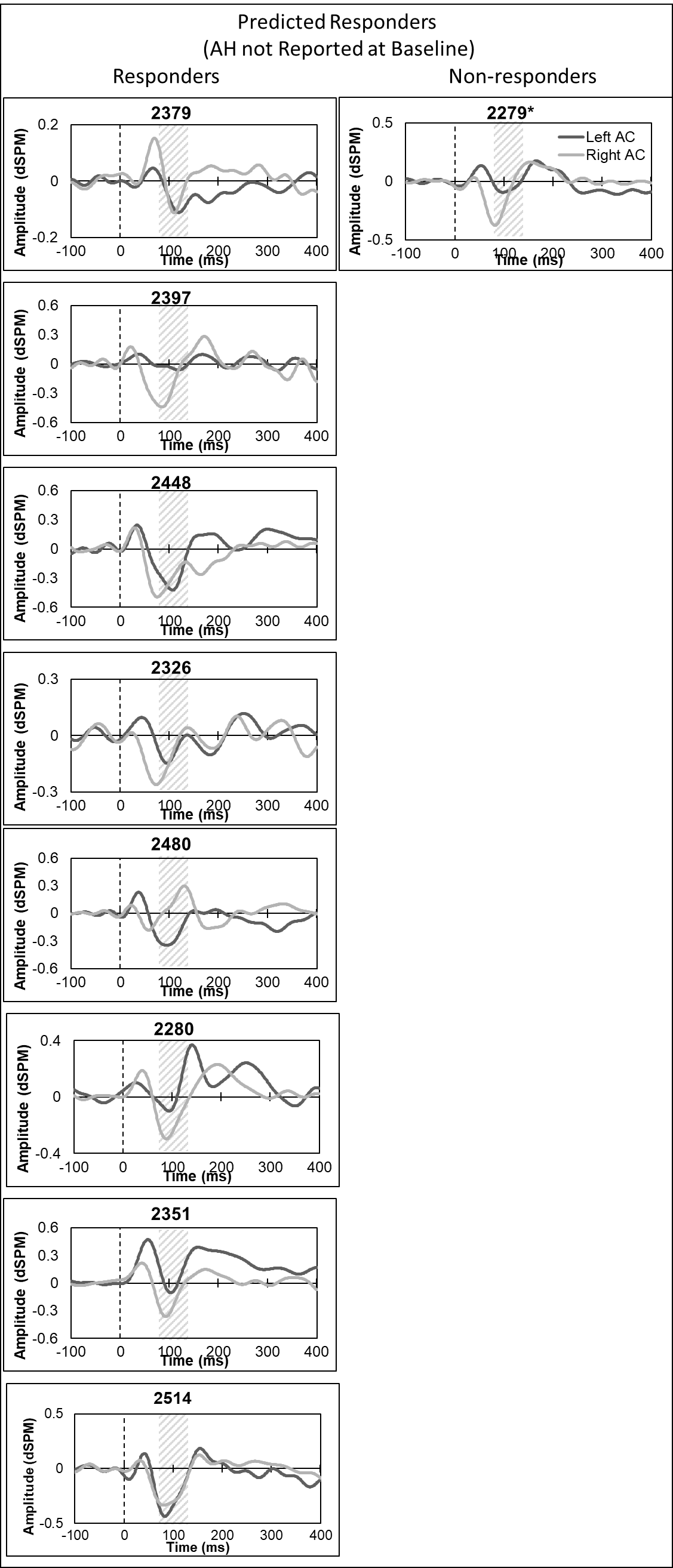


**Figure S4**


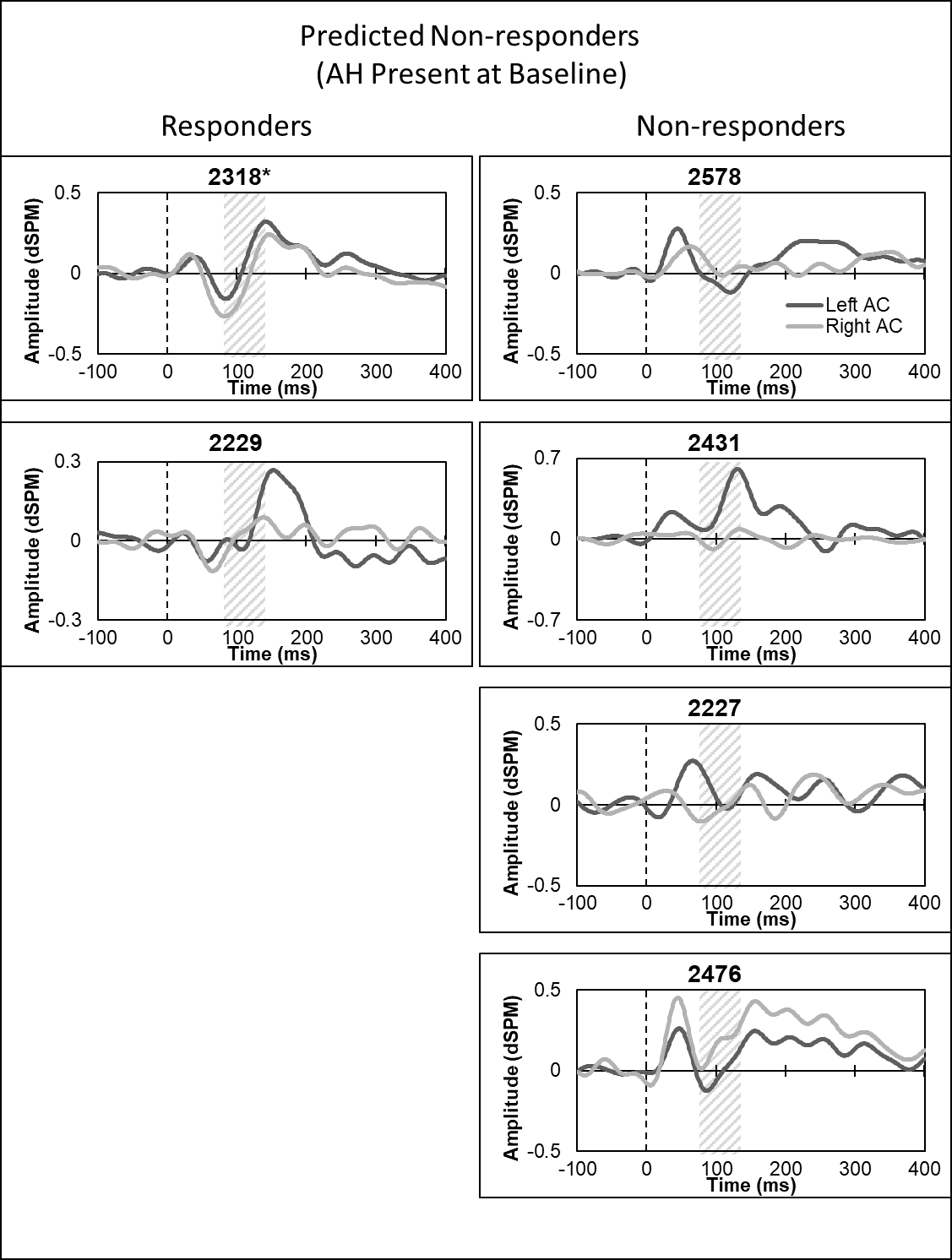


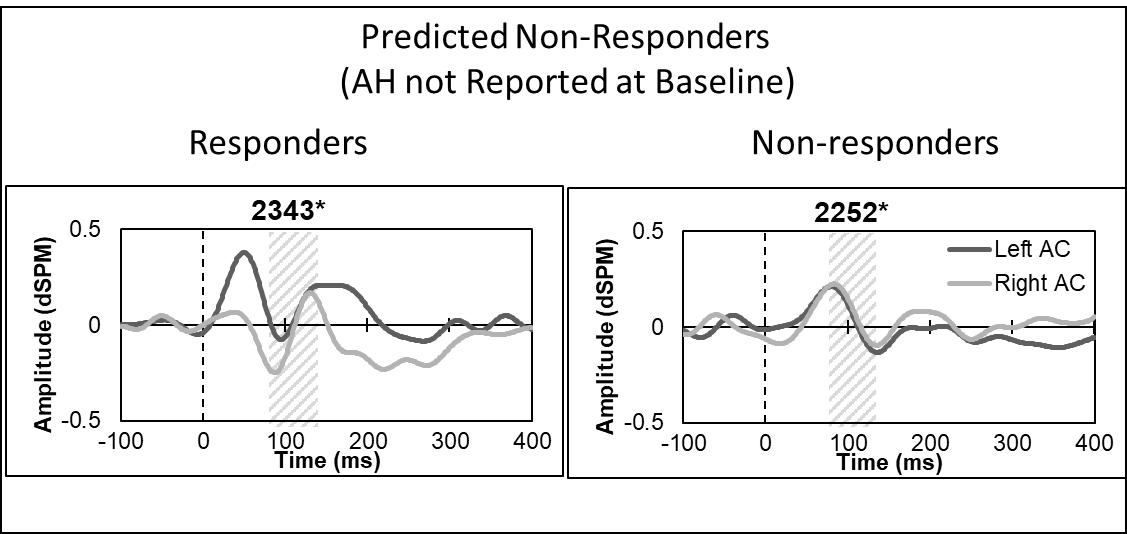


| Age (years) | 22 ± 6 |  |
| --- | --- | --- |
| Sex (M/F) | 19 / 5 |  |
| Race (W / B / A / NA / O) | 6 / 12 / 5 / 0 / 1 |  |
| Participant SES | 29 ± 12 |  |
| Parental SES | 42 ± 14 |  |
| Education (years) | 12 ± 3 |  |
| Duration of Untreated Psychotic Symptoms (DUP, weeks) | 80 ± 69 |  |
| Duration of Untreated Active Psychosis (DAP, weeks) | 35 ± 37 |  |
| WASI IQ | 99 ± 13 |  |
| Verbal IQ | 48 ± 9 |  |
| Performance IQ | 50 ± 9 |  |

**Table S1.** Participant Characteristics. Descriptive statistics are reported for measures obtained at study enrollment.

|  | Mean ± SD | |  |  |
| --- | --- | --- | --- | --- |
|  | T1 | T2 | Δ_(_*_T2-T1)_* | *p-value* |
| Age (years) | 22.9 ± 4.6 | 23.5 ± 4.6 | 0.5 ± 0.1 | **<0.001** |
| MCCB  Processing speed | 35.5 ± 11.2 | 37.5 ± 11.7 | 1.6 ± 10.1 | 0.452 |
| Attention | 33.7 ± 11.1 | 34.6 ± 13.7 | -0.0 ± 11.2 | 0.985 |
| Working memory | 37.4 ± 11.3 | 40.9 ± 9.8 | 2.5 ± 10.2 | 0.250 |
| Verbal learning | 39.1 ± 8.4 | 40.6 ± 7.9 | 1.1 ± 7.1 | 0.469 |
| Visual learning | 36.4 ± 13.7 | 38.3 ± 14.7 | 2.0 ± 11.3 | 0.396 |
| Reasoning | 39.6 ± 11.2 | 42.7 ± 11.8 | 2.3 ± 9.5 | 0.250 |
| Social cognition | 40.0 ± 13.7 | 43.4 ± 14.4 | 2.9 ± 11.7 | 0.244 |
| Total | 29.5 ± 14.8 | 32.6 ± 15.0 | 2.2 ± 9.6 | 0.288 |
| Symptoms  PANSS  General | 41.3 ± 8.2 | 31.4 ± 7.4 | -9.6 ± 6.5 | **<0.001** |
| Negative | 19.2 ± 7.0 | 16.4 ± 6.4 | -2.7 ± 4.1 | **0.005** |
| Positive | 20.9 ± 5.3 | 14.5 ± 5.4 | -6.4 ± 5.8 | **0.001** |
| Total | 81.3 ± 16.8 | 62.1 ± 15.5 | -19.0 ± 13.5 | **0.001** |
| PSYRATS  AVH – Physical | 1.30 ± 1.31 | 0.52 ± 0.87 | -0.8 ± 1.3 | **0.007** |
| AVH – Emotional | 1.26 ± 1.32 | 0.36 ± 0.70 | -0.9 ± 1.4 | **0.004** |
| AVH – Cognitive | 1.25 ± 1.29 | 0.47 ± 0.87 | -0.8± 1.3 | **0.009** |
| AVH – Total | 14.0 ± 14.0 | 5.0 ± 8.4 | -9.0 ± 13.8 | **0.004** |
| Delusions | 12.9 ± 7.1 | 5.2 ± 5.5 | -7.7 ± 5.8 | **<0.001** |
| Medication*  Current Dosage | 255.4 ± 282.9 | 296.4 ± 245.0 | 41.0 ± 331.2 | 0.432 |
| Lifetime | 5980 ± 8070 | 73121 ± 60971 | 67140 ± 59648 | **<0.001** |
| # Medicated | 19 | 20 |  |  |
| *Risperidone* | 11 | 9 |  |  |
| *Haloperidol* | 2 | 1 |  |  |
| *Olanzapine* | 0 | 2 |  |  |
| *Aripriprazole* | 4 | 6 |  |  |
| *Paliperidone* | 3 | 4 |  |  |
| *Quetiapine* | 1 | 0 |  |  |
| *Lurasidone* | 0 | 1 |  |  |

* Medication dosages are reported in Cpz. equivalent dose (mg) for medicated participants only.

**Table S2.** Measures of Cognitive and Psychotic Symptom Severity. Descriptive and inferential statistics are reported for first-episode schizophrenia subjects (FESz) at study enrollment (T1) and approximately 6-months later (T2), as well as the difference between the two. Significant p-values are bolded. All other differences are non-significant (*p*>0.05).

| IV(s) | B | SE(B) | β | r_partial_ | R^2^ | ΔR^2^ |
| --- | --- | --- | --- | --- | --- | --- |
| **Step 1**: Baseline Symptoms | -0.58 | 0.20 | -0.53 | -.53** | 0.28** |  |
|  |  |  |  |  |  |  |
| **Step 2**: Baseline Symptoms | -0.51 | 0.17 | -0.47 | -.55** |  |  |
| M100 Amplitude *(dSPM)* | 23.29 | 7.49 | 0.48 | .56** | 0.51** | 0.23** |
|  |  |  |  |  |  |  |
| Baseline Symptoms | -0.60 | 0.17 | -0.55 | -.62** |  |  |
| log(DAP) | 5.93 | 1.93 | 0.48 | .56** | 0.50** | 0.22** |
|  |  |  |  |  |  |  |
| **Step 3**: Baseline Symptoms | -0.55 | 0.16 | -0.50 | -.61** |  |  |
| log(DAP) | 4.00 | 2.10 | 0.33 | .39* |  |  |
| M100 Amplitude *(dSPM)* | 16.62 | 0.46 | 0.34 | .41* | 0.59** | 0.09* |

**Table S3. Sequential Regression of M100 amplitude and DAP on Positive Symptom Change.** Asterisks represent statistical significance (**p*<0.05; ***p*<0.01).

| IV(s) | B | SE(B) | β | r_partial_ | R^2^ | ΔR^2^ |
| --- | --- | --- | --- | --- | --- | --- |
| **Step 1**: Baseline Symptoms | -0.54 | 0.13 | -0.65 | -.65** | 0.43** |  |
|  |  |  |  |  |  |  |
| **Step 2**: Baseline Symptoms | -0.49 | 0.13 | -0.59 | -.64** |  |  |
| M100 Amplitude *(dSPM)* | 14.41 | 7.75 | 0.29 | .38^†^ | 0.51** | 0.08 |
|  |  |  |  |  |  |  |
| Baseline Symptoms | -0.56 | 0.25 | -0.67 | .72** |  |  |
| log(DAP) | 4.89 | 1.82 | 0.38 | .51* | 0.57** | 0.15* |
|  |  |  |  |  |  |  |
| **Step 3**: Baseline Symptoms | -0.53 | 0.12 | -0.65 | -.69** |  |  |
| log(DAP) | 4.10 | 2.15 | 0.32 | .39^†^ |  |  |
| M100 Amplitude *(dSPM)* | 5.95 | 8.54 | 0.12 | .15 | 0.58** | 0.01 |

**Table S4. Sequential Regression of M100 amplitude and DAP on Delusion Change.** Asterisks represent statistical significance (^†^*p*<0.1; **p*<0.05; ***p*<0.01). Asterisks are not shown for beta statistics, as p-values for these statistics are identical to partial correlation statistics.

| IV(s) | B | SE(B) | β | r_partial_ | R^2^ | ΔR^2^ |
| --- | --- | --- | --- | --- | --- | --- |
| **Step 1**: Baseline Symptoms | -0.81 | 0.13 | -0.82 | -.82** | 0.66** |  |
|  |  |  |  |  |  |  |
| **Step 2**: Baseline Symptoms | -0.83 | 0.10 | -0.83 | -.87** |  |  |
| M100 Amplitude *(dSPM)* | 39.23 | 11.98 | 0.34 | .58** | 0.79** | 0.13** |
|  |  |  |  |  |  |  |
| Baseline Symptoms | -0.93 | 0.11 | -0.93 | -.86** |  |  |
| log(DAP) | 9.11 | 3.61 | 0.30 | .48* | 0.74** | 0.08* |
|  |  |  |  |  |  |  |
| **Step 3**: Baseline Symptoms | -0.88 | 0.11 | -0.89 | -.87** |  |  |
| log(DAP) | 4.71 | 3.87 | 0.16 | .26 |  |  |
| M100 Amplitude *(dSPM)* | 30.56 | 13.81 | 0.26 | .45* | 0.80** | 0.06* |

**Table S5. Sequential Regression of M100 amplitude and DAP on AH change.** Asterisks represent statistical significance (**p*<0.05; ***p*<0.01). Asterisks are not shown for beta statistics, as p-values for these statistics are identical to partial correlation statistics.
